# Phase II Study of Ipilimumab in Men With Metastatic Prostate Cancer With an Incomplete Response to Androgen Deprivation Therapy

**DOI:** 10.3389/fonc.2020.01381

**Published:** 2020-08-07

**Authors:** Julie N. Graff, Mark N. Stein, Rishi Surana, Luai Al Rabadi, Eric Liu, Lawrence Fong, Shawna Bailey, Emile Latour, Timothy A. Newby, Amy E. Moran, Tomasz M. Beer

**Affiliations:** ^1^VA Portland Health Care System, Portland, OR, United States; ^2^Knight Cancer Institute, Oregon Health & Science University, Portland, OR, United States; ^3^Columbia University Medical Center, New York, NY, United States; ^4^MD Anderson, Houston, TX, United States; ^5^Division of Hematology/Oncology, Department of Medicine, University of California, San Francisco, San Francisco, CA, United States

**Keywords:** prostate cancer, immunotherapy, CTLA-4, metastatic, checkpoint inhibitor

## Abstract

**Background:** Phase 3 studies of immune checkpoint inhibitors have not shown a survival benefit in prostate cancer, but some patients have a profound anticancer response.

**Patients and Methods:** We evaluated the efficacy of the CTLA-4 targeted agent, ipilimumab, in metastatic prostate cancer patients who had an incomplete biochemical response to initial androgen deprivation therapy (ADT) alone. Ten patients were enrolled, each treated with ipilimumab 10 mg/kg (every 3 weeks for up to 4 doses) with maintenance ipilimumab every 12 weeks for non-progressing patients. The primary endpoint was proportion of patients with an undetectable PSA. The total sample size was 30 patients, but there was an interim analysis planned at 10 for futility. If none of the 10 patients achieved an undetectable PSA, the study would be halted.

**Results:** The study was halted at the interim analysis as none of the 10 patients achieved the primary endpoint, but 30% of patients demonstrated a >50% reduction in PSA, with one patient achieving a >90% reduction in PSA. Peripheral blood mononuclear cells (PBMC) examined by mass cytometry showed that patients with clinical responses had an increase in effector memory T-cell subsets as well as an increase in T-cell expression of T-bet, suggesting induction of a Th1 response.

**Conclusions:** This study provides further evidence that ipilimumab has activity in some patients with prostate cancer and provides further rationale for the development of future studies aimed at identifying a subset of patients with CPRC that are more likely to derive a benefit from treatment with ipilimumab.

**Implications for Practice:** There is insufficient evidence to use ipilimumab in prostate cancer in routine practice.

**Trial Registration:** ClinicalTrials.gov, NCT01498978. Registered 26 December 2011. https://www.clinicaltrials.gov/ct2/show/NCT01498978?term=julie+graff&rank=3.

## Background

Prostate cancer is currently the second leading cause of cancer deaths in men with more than 33,000 deaths in the United States from prostate cancer expected in 2020 (1). The backbone of therapy for incurable prostate cancer remains androgen deprivation therapy (ADT) with the degree of initial response to ADT strongly correlating with survival. Specifically, those patients who achieve a prostate specific antigen (PSA) level ≤0.2 ng/ml after 6–7 months of ADT have a significantly longer survival compared to those who had PSA nadir >0.2 ng/ml (2). Treatment with ADT, however, is not curative, and most patients will eventually progress to develop metastatic castration-resistant prostate cancer (mCRPC). One approach to address this has been intensification of primary therapy through the addition of chemotherapy or more potent androgen signaling inhibitors (3–8). Such approaches delay, but do not prevent, progression to metastatic CRPC. Thus, there is a need to identify patients at highest risk for developing metastatic disease and to develop treatment modalities that delay progression to mCRPC.

While immunotherapy with checkpoint inhibitors has heralded a new era of cancer treatment and revolutionized the treatment of multiple malignancies including metastatic melanoma and renal cell carcinoma, results in prostate cancer have been equivocal (9–12). Ipilimumab, is a first-in-class fully human monoclonal antibody that binds to cytotoxic T-lymphocyte antigen 4 (CTLA-4) and blocks inhibitory signals expressed on activated T-cells and depletes intratumoral T regulatory cells, promoting anti-tumor activity (13, 14). Results from two phase III trials, published after our study began, demonstrated antitumor activity, and increased progression free survival (PFS) in patients with mCRPC treated with ipilimumab compared to placebo without an improvement in overall survival (OS) (11, 12). Despite these results, we have reported on some exceptional responders to immunotherapy, including one patient with mCRPC treated with ipilimumab who had a profound and durable anti-cancer response, suggesting that there may be a subset of patients very well-suited for checkpoint inhibitors (15).

Our study tested the hypothesis that treatment with ipilimumab in patients with an incomplete response to ADT (PSA >0.2 ng/ml) could lead to complete PSA response and improved survival.

## Methods

### Patient Selection

This single arm, dual center, phase II trial enrolled men ≥18 years of age, with an Eastern Cooperative Oncology Group (ECOG) performance status ≤1, who had histologically confirmed adenocarcinoma of the prostate, castrate levels of testosterone (<50 ng/mL), and a PSA level of >0.2 ng/mL after 6–18 months of ADT utilizing a luteinizing hormone-releasing hormone (LHRH) agonist/antagonist with or without the use of an antiandrogen. ADT was continued throughout the study. If an anti-androgen were stopped prior to enrollment, then it had to be stopped 4 weeks prior to enrollment for nilutamide and flutamide and 6 weeks prior for bicalutamide to ensure an appropriate washout period. All patients had radiographic evidence of distant or regional metastasis at the time of enrollment as detected by computed tomography and/or Technetium-99 bone scan.

Exclusion criteria included prior treatment with ipilimumab or any other CTLA-4 targeting agent (e.g., CD137 agonist) or concomitant therapy with any non-study immunomodulatory agent, radiation therapy to any area of the body within 28 days of enrollment, other active malignancies or autoimmune disorders, leucopenia, neutropenia, platelets <50 × 10^3^/uL, hemoglobin <8 g/dL, creatinine >3.0 × ULN, AST/ALT >2.5 × ULN.

Study sites participating in this study included Oregon Health and Science University (OHSU) and Rutgers Cancer Institute of New Jersey (CINJ). The trial was IRB approved and registered on clinicaltrials.gov (NCT01498978) prior to subject recruitment.

### Study Design

The study design consisted of 4 phases. In the induction phase, all patients received up to four doses of ipilimumab 10 mg/kg IV (administered over 90 min) every 3 weeks. Patients then entered a follow up phase where they were monitored for PSA or radiographic progression. If progression did not occur, patients entered the maintenance phase of the study with the possibility four additional doses of ipilimumab occurring every 12 weeks. The final phase consisted of active follow up of patients until the time of radiographic and/or PSA progression.

The primary endpoint of the study was the proportion of patients who achieve an undetectable PSA (<0.2 ng/mL) after initiation of ipilimumab. Secondary endpoints included time to PSA progression, time to radiographic progression, time to progression by any clinical assessment, time to death from any cause, and maximum percent reduction in PSA.

PSA response was measured using Prostate Cancer Clinical Trials Working Group 2 (PCWG2) 2007 definitions with progression defined as a PSA increase of ≥25% and at least 2 ng/mL from baseline or nadir PSA (confirmed by a second measurement at least 3 weeks later) (16). PSA assessment occurred every 3 weeks during the first 4 cycles and every 6 weeks during weeks 12–24, then every 3 months during the follow-up phase of the study. Radiographic assessments were conducted every 12 weeks via bone scan and a computed tomography (CT) scans of the chest, abdomen, and pelvis using a modified version of RECIST (mRECIST) based on PCWG2 (16). To account for potential “tumor flares,” patients who demonstrated tumor progression or lack of laboratory parameter response prior to week 12 but without rapid clinical deterioration were allowed to continue to be treated with ipilimumab.

### Correlative Endpoints

Serum and PBMCs were collected on day 1 of each cycle and cryopreserved. Samples were then thawed, barcoded, and stained with mass-labeled antibodies. These samples were then washed and data acquired on a mass cytometer (Helios, Fluidigm). Statistical Scaffold was used to generate clustering maps based on marker similarities (to determine cell types) and maps them onto a grid based on manually gated landmark populations (17). Gating strategy was as follows: singlets, live, CD45+CD61-CD235ab–, CD3+CD19– (T cells), CD3+TCR+ (T cells), CD3+CD56 (NK T cells), CD3+CD4+ (CD4 T cells), CD3+CD56-CD8a+ (CD8 T cells), CD3+CD56–TCR–CD8+CD45RO+CD127+ (CD8 Central Memory), CD3+CD56–TCR–CD8+CD45RO+ (CD8 Effector Memory), CD3+CD56–TCR–CD8+CD45RA+CD127+ (CD8 Naïve), CD3+CD56–TCR–CD4+CD25^hi^Foxp3+ (regulatory T cells), CD3+CD4+CD56–TCR–Foxp3–CD45RO+CD127+ (CD4 Central Memory), CD3+CD4+CD56–TCR–Foxp3–CD45RA+CD127+ (CD4 Naïve). Clusters were assigned vectors associated with the average median value of defined protein markers (to evaluate functional status of each cell type) and edges, which are defined as similarity between vectors to produce graphs which show the relationships between different clusters. Cluster frequencies and boolean expression for certain markers for each cluster were passed through the Significance Across Microarrays algorithm and results were formulated into the Scaffold maps for visualization (github.com/nolanlab/scaffold).

### Statistics

Ten patients were initially accrued using the Simon two-stage design for phase II trials. At least two patients with an undetectable PSA were required within the initial 10 patient cohort to continue to stage II of the patient accrual with a total planned enrollment of 30 patients. The Kaplan-Meier method was used to estimate median time-to-event outcomes and to generate survival curves.

## Results

**Figure 1:**
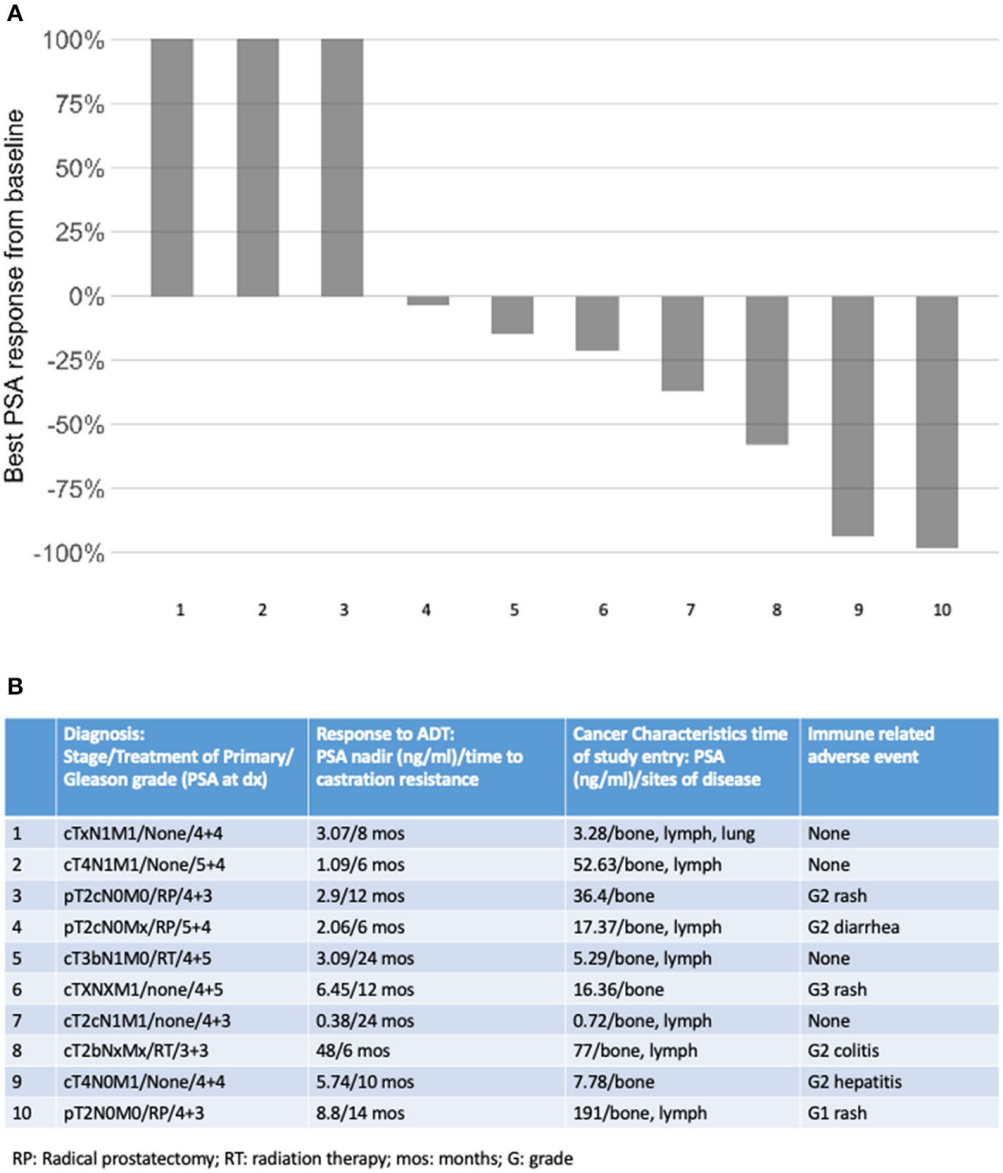
**(A)** Best PSA response on study; **(B)** tumor and patient factors according to cancer response.

**Figure 2:**
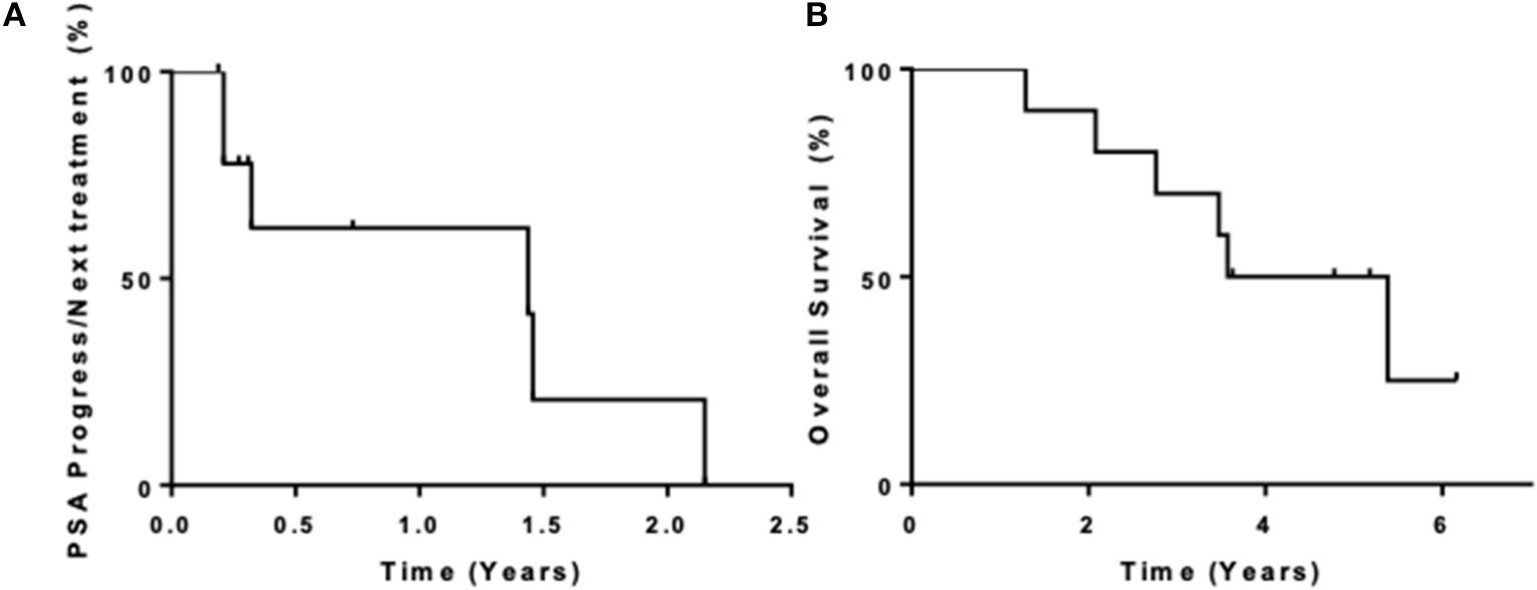
**(A)** PSA progression and time to next therapy; **(B)** overall survival.

Ten patients were enrolled in this study from September 2012 to June 2015. The data cutoff used in this analysis is May 9, 2019. The median age of patients at enrollment was 65 years with a median PSA of 14 (Table 1). Prior to this study, 6 participants had nadired to a PSA 0.2–4 ng/ml, and 4 never got to 4 ng/ml. All patients had radiological evidence of metastases at enrollment. A median of 3 cycles of ipilimumab were administered with two patients completing 2 cycles of ipilimumab, four patients completing 3 cycles, two patients completing 4 cycles, and two patients receiving the full course of 8 cycles. None of the patients enrolled reached the primary endpoint of complete biochemical response evidence by undetectable PSA (<0.2 mg/mL). Thus, per protocol, this study did not move to stage II. Although the primary endpoint was not reached, there was evidence of clinical responses. Three of the ten (30%) experienced a >50% decline in PSA level, two of these demonstrating a decline of more than 90% and one of these a decline of >98% (Figure 1). The median time to PSA progression was 17.2 months (95% CI 2.53 to NR) (Figure 2A). Median time to radiographic progression was not reached. However, all patients eventually demonstrated biochemical progression (Figure 2A). Three out of 10 (30%) remain alive with follow-up ranging from 44.1 to 58.0 months and with a median overall survival of 53.6 (95% CI: 15.4 to NR) (Figure 2B). Median OS was 42.2 months in patients with a PSA nadir prior to study of 0.2–4.0 ng/ml and 64.5 months in patients with a PSA that did not nadir to 4 ng/ml or lower. None were related to immune-mediated complications or treatment-related side effects.

**Table 1:** Baseline Characteristics (ADT, androgen deprivation therapy; mHNPC, metastatic hormone naïve prostate cancer).

**Enrolled**	**10**
**Age**	
Median	64.5 years (56–69)
**Race**	
Caucasian	9
Asian	1
**ECOG**	
0	7
1	3
**Stage at initial presentation**	
Localized, node negative	1
Localized, node positive	4
Distant metastatic disease	5
**Gleason score**	
6	1
7	3
8–10	6
**Prior treatment to the primary**	
Surgery	3
Radiation	1
None	6
**Sites of metastatic disease**	
Bone Only	3
Lymph only	1
Bone and lymph	6
Visceral	0
**PSA nadir on ADT**	
0.2–4 ng/ml	6
>4 ng/ml	4
**PSA at cycle 1**	
0.2–4 ng/ml	2
>4 ng/ml	8
Chemotherapy for mHNPC	1

Adverse events were common and consistent with previous ipilimumab experience (Table 2). Overall, six patients (60%) developed one or more adverse events. One patient developed multiple immune-related adverse events (irAEs) most prominently grade 2 hepatitis and rash that required treatment with steroids and eventually with infliximab. Notably, this patient also had the largest absolute decline in PSA mentioned above. Another patient developed grade 2 adrenal insufficiency requiring steroid replacement while another experienced an acute kidney injury for which cycle 3 was withheld. Four patients developed two or more irAEs. No patients in this study experienced a grade 4 or higher adverse event (Table 2). There was not a significant association between irAEs and response.

**Table 2:** Adverse events (AE): (A) non-immune related; (B) immune related (AST, Aspartate Aminotransferase; ALT, Alanine Aminotransferase).

**Adverse event**	**Grade 1**	**Grade 2**	**Grade 3**
**(A)**			
**Number of patients**			
Pruritis	5		
Diarrhea	5	4	
Rash	1	4	1
Fatigue	2	2	
Fever	3		
Myalgias (leg cramps)	4		
Dry skin	1		
Hypotension	1		1
Vomiting		1	
Increased creatinine		1	
Flu like symptoms		1	
Elevated AST	1	1	
Elevated ALT	1	1	
Elevated alkaline phosphatase	1		
Colitis			1
Adrenal insufficiency		1	
Nausea	1	1	
Chills	1		
Anorexia		1	
Bloating		1	
Malaise	1		
Hyponatremia	1		
Hypokalemia	1		
Dupuytren's contracture	1		
**(B)**			
**Immune-related adverse events**	**Grade (number of patients if more than 1)**		
Colitis	3		
Maculopapular rash	1,2,3		
Diarrhea	1(2), 2		
Adrenal insufficiency	2		
Pruritis	1		
Fever	1		
Elevated AST	2		
Elevated ALT	2		
Elevated alkaline phosphatase	1		

**Figure 3:**
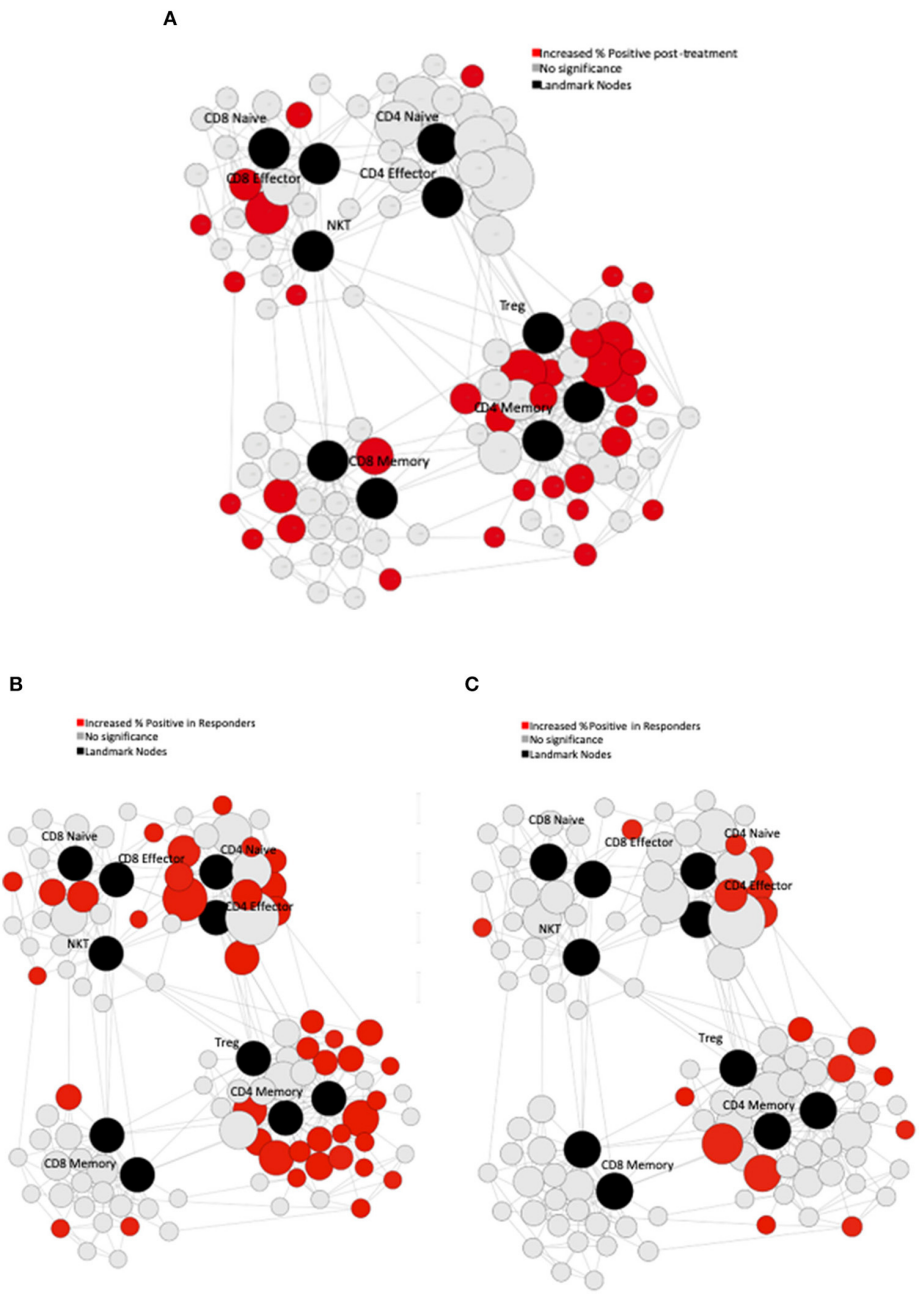
Immune Characteristics. CyTOF assessment of peripheral blood mononuclear cells (PBMCs) from treated patients. **(A)** Pre-treatment PBMCs were compared to post-treatment PBMCs using Statistical Scaffold analysis. Landmark nodes are denoted in black and serve as reference points representing pre-determined cell subsets. Sample cluster sizes generated from distinct cell populations surround the landmark nodes and edges connect clusters to one another based on similarity to guide the development of Scaffold maps. Sample cluster sizes are scaled according to the population of each cluster. Clusters with statistically significant increase in the % of cells that are Ki-67 positive in post-treatment samples are denoted in red (*q* < 0.05). **(B)** Pre-treatment PBMCs in patients with PSA responses were compared to the PSA non-responders with Statistical Scaffold. Clusters with statistically significant increase in t-bet pre-treatment are denoted in red (*q* < 0.05). **(C)** Clusters with statistically significant increase in PD-1 pre-treatment are denoted in red (*q* < 0.05).

Serial PBMC were available for eight of the study subjects. We performed immune phenotyping by mass cytometry from the patients to assess for treatment induced effects. In response to treatment, there was an increase in Ki-67 across multiple PBMC subsets when comparing pre-treatment to post-treatment samples (Figure 3A). In exploratory analyses, we compared the immunologic profiles in patients with PSA responses (>50% declines) vs. non-responders. In the pre-treatment PBMCs, our analysis revealed that patients with PSA responses had significantly higher T-bet and PD-1 expression in CD4 T-cells (Figures 3B,C, respectively). Finally, we observed an overall increase in the percentage of T-bet positive T-cells suggesting expansion of Th1 cells was favored in patients who had PSA declines vs. those who did not (Figure 3C).

## Discussion

This study examined the effect of CTLA-4 blockade in men with metastatic prostate cancer who did not achieve an undetectable PSA, and it failed to meet its primary endpoint. Since this study was designed in 2009, management of metastatic prostate cancer has changed dramatically. First, we have five new therapies that prolong survival in mCRPC. Second, we now treat newly diagnosed metastatic prostate cancer more aggressively. Third, we have more information about how checkpoint inhibitors perform in mCRPC, and we know that single-agent therapy may be inferior to combination approaches. Fourth, we are more attuned to the mutational landscape of mCRPC and are pairing patients to treatments based on our findings (e.g., mismatch repair deficiency and DNA repair defects). Therefore, it is possible outcomes would be different if we used this information to enroll for this trial today.

Since 2009, there have been 5 trials in mCRPC with ipilimumab (Table 3). A phase I/II study of patients receiving ipilimumab (10 mg/kg) with external beam radiation therapy showed that chemotherapy naïve patients may have more PSA responses (6 responders out of 23 patients, 26%) compared to chemotherapy experienced patients (2 responders out of 27 patients, 7.4%) (19). Similarly, a phase III study randomized 799 patients with mCRPC previously treated with chemotherapy to either ipilimumab plus radiation vs. placebo plus radiation. Although the primary endpoint of improved OS in the ipilimumab treated patients was not met, the ipilimumab arm had a superior progression free survival (PFS) (4.0 vs. 3.1 months) (11). A *post-hoc* analysis suggested a benefit with ipilimumab in patients with more favorable prognostic factors, specifically alkaline phosphatase ≤1.5 times the upper limit of normal, hemoglobin 11 g/dL and the absence of visceral metastases (11). In a second phase III study, using chemotherapy naïve patients who were either asymptomatic or minimally symptomatic, those with visceral metastases were excluded. This study randomized 602 patients to receive ipilimumab 10 mg/kg every 3 weeks for four doses or placebo. No difference in OS was observed, but patients treated with ipilimumab had a longer PFS (5.6 vs. 3.8 months, 95% CI, 0.55–0.81, HR 0.67) and were more likely to have a PSA response (23 vs. 8%) (12). Additionally, there is a study with finite ADT with ipilimumab in men with metastatic castration sensitive disease; 27 patients received 8 months of ADT with ipilimumab before the early termination of the study due to grade 3 irAEs in more than 40% of subjects (22). The 18 patients who did not progress during ADT had their ADT discontinued. The median time to PSA progression was 10.0 months following day 1 of ADT, and there were complete responses in 2 patients. The investigators found that clonal expansion of CD8+ T cells preceded the development of severe irAEs.

**Table 3:** Studies of Ipilimumab in mCRPC and PSA response rates (OS, Overall survival; PFS, Progression-free survival; PSA, Prostate specific antigen).

**Study/Intervention**	**Patients**	**Outcomes**
Phase I: Ipilimumab + Poxviral vaccine Escalating Ipi: 1, 3, 5, 10 mg/kg; 6 or more doses Recombinant fowlpox PSA-Tricom: monthly boosts (18)	Asymptomatic 6 post docetaxel 24 docetaxel naïve	Post docetaxel: 1 (17%) of 6 had a PSA response; median PFS = 2.4 mos (1.5–3.7) Chemotherapy naïve: 14 (58%) of 24 had PSA response; 6 (25%) of 24 had PSA declines >50%; median PFS 5.9 (3.4–8.8)
Phase I/II: Dose escalation study with Ipilimumab +/– radiation (8Gy/lesion) (19)	Asymptomatic, +/– prior treatments	In the 10 mg/kg ipilimumab +/– radiation cohort (*n* = 50), 8 had confirmed PSA decline (6 prior chemo, 2 chemo naïve)
Phase I: Dose escalation study with Ipilimumab (up to 10 mg/kg) + Sargramostim (20)	42 patients, chemotherapy naïve	5 patients experienced > 50% decline in PSA (2 of them in 10 mg/kg ipilimumab cohort)
Phase II: Single-Arm Ipilimumab (3 mg/kg IV every 3 weeks, up to 4 doses) (21)	30 patients with mCRPC	Median PSA PFS: 1.7 mo; median radiographic PFS: 3.0 mo; median OS 24.3 mo
Phase III: Ipilimumab (10 mg/kg) vs. placebo (12)	602 patients, chemotherapy-naïve, asymptomatic (or minimally symptomatic)	Median PFS: 5.6 mo in ipilimumab cohort vs. 3.8 mo in placebo OS: No statistically significant difference between two cohorts
Phase III: Ipilimumab (10 mg/kg) vs. placebo (11)	799 patients, all had prior radiation treatment	Median PFS: 4.0 mo in ipilimumab cohort vs. 3.1 in placebo OS: No statistically significant difference between two cohorts

Although no patient met the primary endpoint of PSA <0.2 ng/dl, 30% of patients achieved a >50% decline in PSA while on study demonstrating antitumor activity in this patient population. The primary endpoint chosen for this trial is unusual, although there is rationale to using it. If this study had been powered with a different primary endpoint in mind, it might have yielded more clinically informative data. In this small study, those who had a PSA nadir of 0.2–4 ng/ml did not do better than those who never reached 4 ng/ml. A key determinant in identifying potential responders to checkpoint-inhibition, and what likely drove the lack of complete PSA response observed in this study, is both intra- and inter-tumor heterogeneity that likely drives systemic anti-tumor immune responses. Treatment with anti-CTLA-4 resulted in alteration of the phenotype of effector T cells. Specifically, higher percentage PD-1+ CD4 T cells correlated with clinical response, which is consistent with prior studies demonstrating that pre-existing CD4 T cells expressing PD-1 correlated with overall survival (23). Furthermore, the data suggest that a higher abundance of T-bet positive Th1 T cells correlated with PSA response both before and after treatment, while there seemed to be an inverse relationship between the effector and central memory T-cells; multi-dimension analysis tools demonstrated that a higher frequency of CD4 effector memory cells correlated with a PSA response, whereas a high proportion of CD4 central memory cells correlated with no PSA response. These data expand on existing literature that suggests that patients with a specified pre-treatment immunologic signature may be more likely to respond to treatment with checkpoint inhibition (24). Clearly there needs to be prospective study with tumor biopsies to determine if these changes in the peripheral blood are reflected in the tumor microenvironment.

Relative to other genitourinary malignancies, trials investigating single-agent checkpoint inhibitor immunotherapy in prostate cancer have been disappointing. However, ongoing work combining checkpoint inhibitor therapy with other known active prostate cancer agents is tantalizing. In one phase II study, the PD-1 inhibitor pembrolizumab was combined with enzalutamide in 28 patients with metastatic castrate resistant prostate cancer (mCRPC) who were progressing on enzalutamide (25). Five of 28 patients (18%) reached a PSA of 0.2 ng/dl. Similarly, in another phase I/II study, 17 patients with mCRPC who had progressed on enzalutamide and/or abiraterone were treated with durvalumab plus the poly (ADP-ribose) polymerase inhibitor, olaparib, until disease progression, or unacceptable toxicity (26). Notably in the second study, seven patients had deficiencies in homologous recombination (BRCA2), for which olaparib is a treatment, and this makes determination of relative contribution of the checkpoint inhibitor difficult. In this study, 9 of 17 (53%) patients had a radiographic and/or PSA response (PSA decline of ≥50%). In addition, the efficacy of combination immunotherapy with nivolumab and ipilimumab has been evaluated in two clinical trials. The preliminary results of one trial of 78 patients with a minimum of 6 months follow up demonstrated an ORR of 26% (6 of 23) and 10% (3 of 30) among patients that were chemotherapy naïve and those previously treated with taxane-based therapy, respectively (27). The second trial focused on patients with AR-V7 mutations and showed a PSA response in 2/15 (13%) participants (28).

There are trials accruing for men with mCRPC that include ipilimumab. The CheckMate 650 trial is a phase II trial planning to enroll 618 participants, who will be randomized to receive nivolumab with ipilimumab, ipilimumab monotherapy, or cabazitaxel. The primary endpoints are objective response rate and overall progression free survival (NCT02985957). Another randomized phase II study will examine the effects of abiraterone, prednisone, apalutamide with or without ipilimumab (NCT02703623).

## Conclusion

In conclusion, this trial evaluated the utility of early initiation of ipilimumab in patients with an incomplete response to ADT. The primary endpoint of complete biochemical response was not met, but there was evidence of clinical activity of ipilimumab in a subset of trial participants. This is consistent with finding of studies in CRPC and extends those findings into patients at a somewhat earlier point in their disease course. More work is needed to identify a subset of patients with CRPC that will likely benefit from checkpoint inhibition. These studies will likely include assessments of immune response before and after treatment, understanding resistance mechanisms (e.g., upregulation of other checkpoint molecules), understanding the fundamental tumor biology that specifically predisposes some tumors to be susceptible to checkpoint inhibition (e.g., microsatellite instability), and evaluating combination therapy with immunotherapy and other known active agents in prostate cancer.

## Data Availability Statement

The datasets generated for this study are available on request to the corresponding author.

## Ethics Statement

The studies involving human participants were reviewed and approved by OHSU Institutional Review Board. The patients/participants provided their written informed consent to participate in this study. Cancer Institute of New Jersey (CINJ) also had IRB approval. MS was there when he enrolled patients.

## Author Contributions

Trial conceived of and designed by JG and TB. Data collected by JG, MS, SB, TN, and TB. Analysis was performed by JG, MS, RS, LA, ELi, LF, SB, ELa, TN, AM, and TB. Paper written and approved by JG, MS, RS, LA, ELi, LF, SB, ELa, TN, AM, and TB. All authors contributed to the article and approved the submitted version.

## Conflict of Interest

LF and MS have received research funding from Bristol Myers Squibb. The remaining authors declare that the research was conducted in the absence of any commercial or financial relationships that could be construed as a potential conflict of interest.
